# Iliopsoas Abscess (together with Bullet) Resulting from a Firearms Injury

**DOI:** 10.1155/2015/634356

**Published:** 2015-05-14

**Authors:** Yunus Güzel, Sadettin Çiftçi, Ali Özdemir, Mehmet Ali Acar

**Affiliations:** ^1^Department of Orthopaedics and Traumatology, School of Medicine, Ordu University, Campus of Cumhuriyet, Center, 52200 Ordu, Turkey; ^2^Department of Orthopaedics and Traumatology, Selçuklu School of Medicine, Selçuk University, Campus of Alaeddin Keykubat, Selçuklu, 42075 Konya, Turkey

## Abstract

Psoas abscess, which is a rarely encountered infection, is defined as the accumulation of suppurative fluid within the fascia surrounding the psoas and iliac muscles. It is categorised as being primary or secondary. Although there are reports in the literature of secondary psoas abscess from foreign bodies, to the best of our knowledge, this is the first reported case of psoas abscess developing due to a bullet, following a firearms injury. The patient was first seen in the Emergency Department following a firearms injury in the posterolateral lumbar region and as the neurovascular examination was normal, the patient was discharged after 24 hours of observation. One month later, the patient presented again to the polyclinic with a high temperature and back pain. As a result of physical examination and tests, a diagnosis was made of psoas abscess and percutaneous drainage was applied under ultrasonography guidance. The complaints improved but, 10 days later with an increase in pain and indications of infection, open abscess drainage was applied and the bullet was removed. At the 6-month follow-up examination, the patient had no complaints.

## 1. Introduction

Iliopsoas abscess (IPA) is the accumulation of suppurative fluid within the fascia surrounding the iliacus and psoas muscles [1]. Although rarely seen, it can cause life-threatening tables. In the past, it was seen most often as a complication of tuberculosis and although the incidence of tuberculosis has decreased, the frequency of hematogenous IPA has increased [2]. The etiology of IPA is separated into two groups as primary and secondary. Primary IPA is associated with hematogenous* Staphylococcus* bacteremia and secondary IPA develops following infection spreading from surrounding tissues [2, 3]. Secondary IPA may be seen following the spread of gastrointestinal or urinary system infection, discitis, osteomyelitis, septic hip arthritis, or infected hip prosthesis [4, 5]. Abscesses associated with foreign bodies have been mentioned in the literature but there are no reports of a psoas abscess associated with a bullet following a firearms injury. This paper presents the case of an iliopsoas abscess with the etiology from a bullet.

## 2. Case Report 

**Figure 1:**
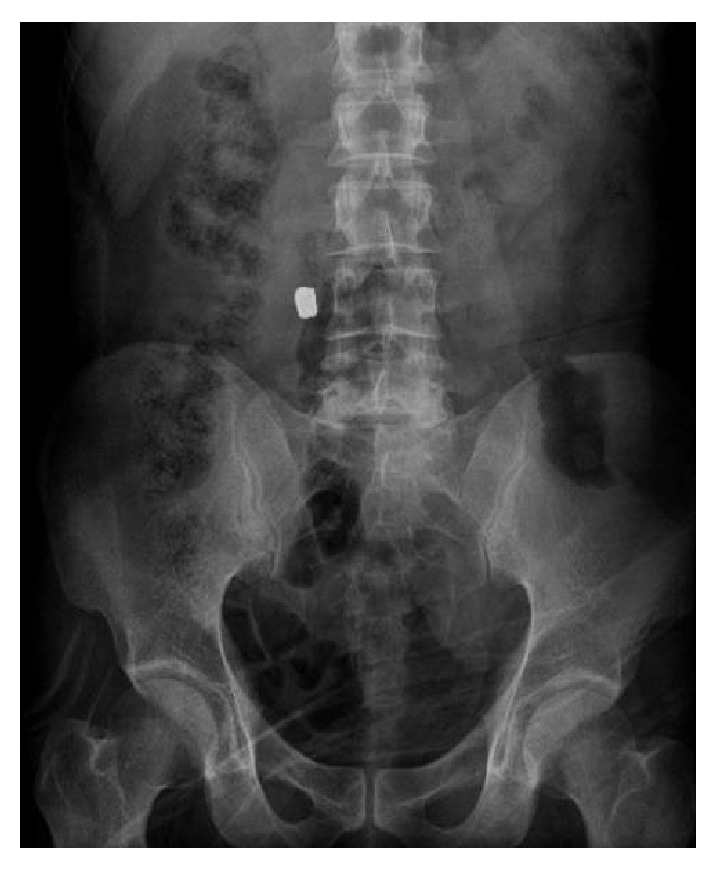
Bullet seen on the anterior-posterior radiograph.

A 37-year-old male presented to the Emergency Department with a firearms injury. In the physical examination, the bullet entrance hole was determined to be between the right spina iliaca anterior superior and the 12th rib in the posterolateral region. The neurovascular examination results were normal. On the direct anterior-posterior radiograph, the bullet was seen on the right side of the lumbar 4th rib (Figure 1). There was no abnormality in the abdominal examination and no internal abdominal injury was determined on the computed tomography (CT) images. After a 24-hour observation period, prophylactic antibiotic treatment (cefazolin sodium 3 × 1 gr i.m) was started and the patient was given a polyclinic follow-up appointment.

**Figure 2:**
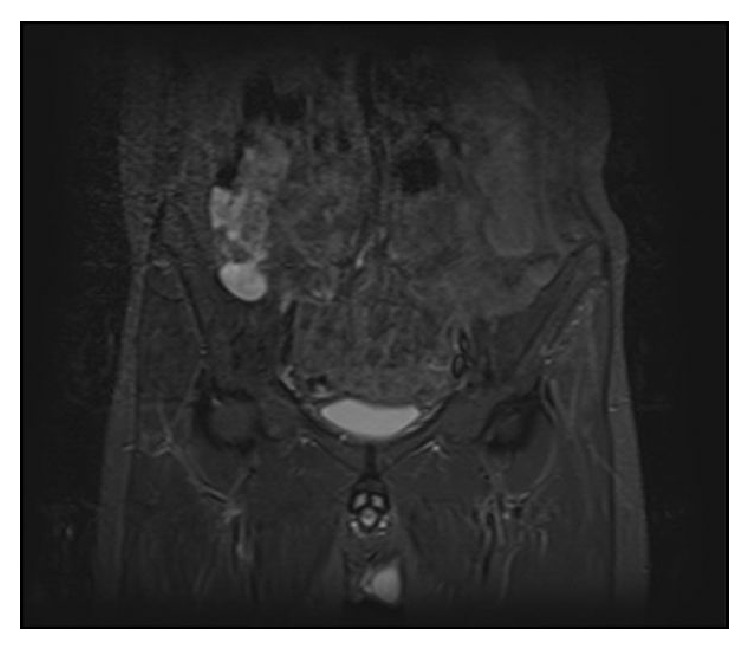
MRI of accumulated suppurative fluid within the iliopsoas fascia.

**Figure 3:**
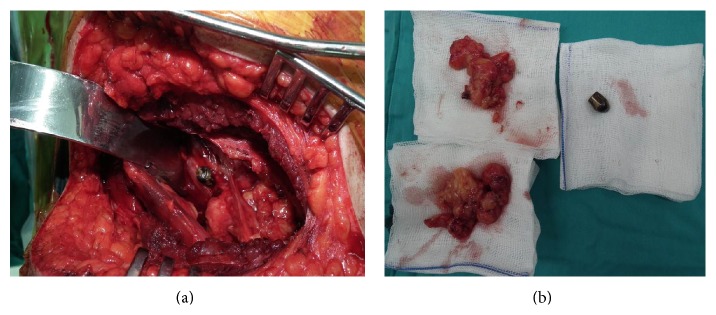
(a) Intraoperative view of the bullet in the iliopsoas. (b) The granulomatous tissue and bullet removed.

At the follow-up examinations, the bullet entrance hole was seen to have closed without any problems and the patient was mobile. However, after 1 month the patient presented again to the Emergency Department with complaints of high temperature and pain in the back and on the right side and from the examination of magnetic resonance imaging (MRI) the diagnosis of IPA was made (Figure 2). Percutaneous drainage was applied under ultrasonography (USG) guidance. As there was production of* Staphylococcus aureus* on the drainage fluid, antibiotic treatment was started with advice of the Infectious Diseases Department. After the percutaneous drainage, the patient experienced relief of the complaints but then presented to the polyclinic 10 days later with the same complaints. In the laboratory test results, the erythrocyte sedimentation rate was 56 and C-reactive protein value was 47 and, on USG, abscess accumulation was again seen. Surgery was planned. The patient was placed in a lateral position and with an anterolateral incision and retroperitoneal approach the iliopsoas fascia was reached, the abscess was drained, culture was taken, and the bullet was removed (Figure 3). At the 6-month follow-up, the patient had no complaints and the infection markers were normal.

## 3. Discussion

Primary IPA and secondary IPA which develop following staphylococcic bacteremia of unknown cause have the same clinical and disease courses. Secondary IPA generally occurs via contamination from the surrounding tissues involved in the urinary or gastrointestinal system [6]. Pyogenic sacroiliitis [7], kidney infection [8], aortic infection [9], and infected hip prosthesis [5] are uncommon etiologies of psoas abscess. A 1991 report in the literature describes a psoas abscess associated with a firearms injury [10]. However, in that study it was concluded that the psoas abscess etiology was from fecal contamination observed on bullet fragments following a firearms injury to the colon. In the case presented here, the abdomen was not affected by the injury and CT examination showed all the internal abdominal organs to be normal. To the best of our knowledge, this is the first case of IPA caused by a firearms injury, which did not involve the abdomen and, as such, has a place in the literature as an uncommon etiology of psoas abscess.

Iliopsoas abscess is a condition which is difficult to diagnose and, if not treated, has a mortality rate of up to 20% [2, 3]. When initial diagnosis is considered, USG and MRI should support the diagnosis and investigation must be made in respect of primary or secondary iliopsoas abscess. A high index of suspicion is necessary for early diagnosis and prompt management. Although USG can show abscess fluid collection, MRI is more effective in respect of determining the etiology [11]. When iliopsoas abscess is being considered, diagnosis is facilitated with the widespread use of CT and MRI and the pathology in surrounding tissues can be shown more effectively for a more accurate prediction of etiology. In addition to the treatment of abscess drainage, antibiotic treatment is effective. However, of the drainage choices, whether the percutaneous drainage or open surgery drainage method is more effective is still a subject of debate [2, 12, 13]. In studies by Hsieh et al. [2], as recurrence was often seen following percutaneous drainage, open drainage was recommended, especially in cases with gas formation. Similarly, open drainage has been recommended if the abscess is multilocular [14]. In the case presented here, recurrence was observed after percutaneous drainage under USG guidance and so open surgery drainage was applied.

Several factors may cause an abscess, such as the relationship of the iliopsoas fascia with the retroperitoneal lymphatic circulation, enriched blood circulation, or the fact that the gastrointestinal tract and the urinary system are adjacent and extend to the pelvis even as far as the hip joint [2]. While the etiology of several abscesses may be clarified with CT and MRI, there may be underlying factors such as intestinal rupture, Crohn's disease, or discitis. In the case presented here, the bullet from a firearms injury caused the abscess without damaging any internal abdominal organ.

## 4. Conclusion

As the patient had no neurovascular injury, he was discharged after a 24-hour observation period and the iliopsoas abscess formation was seen to recur. In firearms injuries seen in the Emergency Department, if the injury is in a region where there is a risk of infection developing, such as the iliopsoas fascia, then even if there is no neurovascular injury, the patient should be followed up in the clinic with broad spectrum antibiotics prophylaxis. In firearms injuries, should every bullet seen in the iliopsoas fascia be removed? This is a separate question, but if it is seen together with an abscess, then rather than percutaneous drainage the method of open surgery drainage and bullet removal should be preferred.
